# Late gadolinium enhancement entropy as a new measure of myocardial tissue heterogeneity for prediction of adverse cardiac events in patients with hypertrophic cardiomyopathy

**DOI:** 10.1186/s13244-023-01479-6

**Published:** 2023-08-21

**Authors:** Zi-Yi Gu, Yu-Fan Qian, Bing-Hua Chen, Chong-Wen Wu, Lei Zhao, Song Xue, Lei Zhao, Lian-Ming Wu, Yong-Yi Wang

**Affiliations:** 1 Department of Cardiovascular Surgery, Ren Ji Hospital, Shanghai Jiao Tong University School of Medicine, Shanghai, 200127 China; 2 Department of Radiology, Ren Ji Hospital, Shanghai Jiao Tong University School of Medicine, Shanghai, 200127 China; 3 Department of Radiology, Beijing Anzhen Hospital, Capital Medical University, Beijing, 100029 China

**Keywords:** Magnetic resonance imaging, Prognosis, Hypertrophic cardiomyopathy

## Abstract

**Objectives:**

Entropy is a new late gadolinium enhanced (LGE) cardiac magnetic resonance (CMR)–derived parameter that is independent of signal intensity thresholds. Entropy can be used to measure myocardial tissue heterogeneity by comparing full pixel points of tissue images. This study investigated the incremental prognostic value of left ventricular (LV) entropy in patients with hypertrophic cardiomyopathy (HCM).

**Methods:**

This study enrolled 337 participants with HCM who underwent 3.0-T CMR. The LV entropy was obtained by calculating the probability distribution of the LV myocardial pixel signal intensities of the LGE sequence. Patients who underwent CMR imaging were followed up for endpoints. The primary endpoint was defined as readmission to the hospital owing to heart failure. The secondary endpoint was the composite of the primary endpoint, sudden cardiac death and non-cardiovascular death.

**Results:**

During the median follow-up of 24 months ± 13 (standard deviation), 43 patients who reached the primary and secondary endpoints had a higher entropy (6.20 ± 0.45, *p* < 0.001). The patients with increased entropy (≥ 5.587) had a higher risk of the primary and secondary endpoints, compared with HCM patients with low entropy (*p* < 0.001 for both). In addition, Cox analysis showed that LV entropy provided significant prognostic value for predicting both primary and secondary endpoints (HR: 1.291 and 1.273, all *p* < 0.001). Addition of LV entropy to the multivariable model improved model performance and risk reclassification (*p* < 0.05).

**Conclusion:**

LV entropy assessed by CMR was an independent predictor of primary and secondary endpoints. LV entropy assessment contributes to improved risk stratification in patients with HCM.

**Critical relevance statement:**

Myocardial heterogeneity reflected by entropy the derived parameter of LGE has prognostic value for adverse events in HCM. The measurement of LV entropy helped to identify patients with HCM who were at risk for heart failure and sudden cardiac death.

**Key points:**

• Left ventricular entropy can reflect myocardial heterogeneity in HCM patients.

• Left ventricular entropy was significantly higher in HCM patients who reached endpoint events.

• Left ventricular entropy helps to predict the occurrence of heart failure and death in HCM patients.

**Graphical Abstract:**

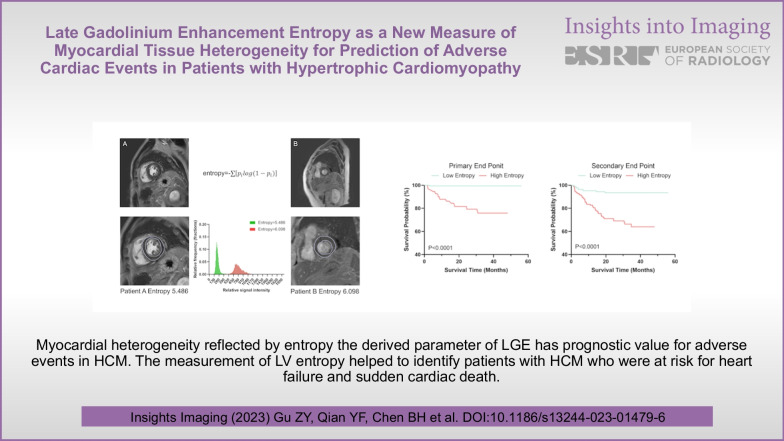

**Supplementary Information:**

The online version contains supplementary material available at 10.1186/s13244-023-01479-6.

## Introduction

Hypertrophic cardiomyopathy (HCM) is a common inherited cardiovascular disease found in one in 500 among the general population [1, 2]. The morphological and functional features of HCM include marked and asymmetric left ventricular (LV) hypertrophy and a non-dilated LV cavity [3]. There is a significant heterogeneity in the clinical phenotypes of patients with HCM, who can be asymptomatic or present with heart failure (HF), arrhythmias, and sudden cardiac death (SCD) [4]. With the use of implantable defibrillators, the incidence of HCM-related SCD has decreased significantly, and HF is becoming an increasingly prominent management challenge for HCM [5]. Various risk factors have been identified for the poor prognosis of HCM patients [6, 7]. However, accurate risk prediction remains inadequate.

Cardiac magnetic resonance (CMR) is particularly suitable for the analysis of phenotypic changes in HCM due to its unique advantages in histological characterization [8–10]. CMR using late gadolinium enhancement (LGE) to detect myocardial fibrosis is one of the most important techniques to non-invasively characterize scar tissue. Recent researches have shown the value of LGE in predicting adverse cardiac events to contribute to risk stratification in clinical practice [14–16]. However, most LGE quantification methods are performed by setting a signal intensity threshold and evaluating a range of pixels above that threshold. There are limitations to the assessment of the overall myocardium, especially the non-enhanced regions [17].

Entropy is a new LGE-derived parameter that is independent of the signal intensity threshold [18, 19]. An image with perfectly homogeneous pixels will have zero entropy. When there is a difference in signal intensity between myocardial tissues, there will be many different pixel values, and thus a higher entropy. According to this principle, entropy can quantify the "complexity" of the entire left ventricular myocardium. Previous studies have found that entropy can reflect myocardial inhomogeneous remodeling in dilated cardiomyopathy and post-infarction [20, 21]. Considering that HCM is characterized by diffuse histopathological abnormalities [22], we hypothesized that LGE entropy measurements could better reflect the myocardial heterogeneity of HCM patients. Therefore, in this study, we investigated whether LV entropy is associated with poor prognosis in patients with HCM and assessed the predictive value of entropy as a risk stratification.

## Methods

### Study population

**Fig. 1:**
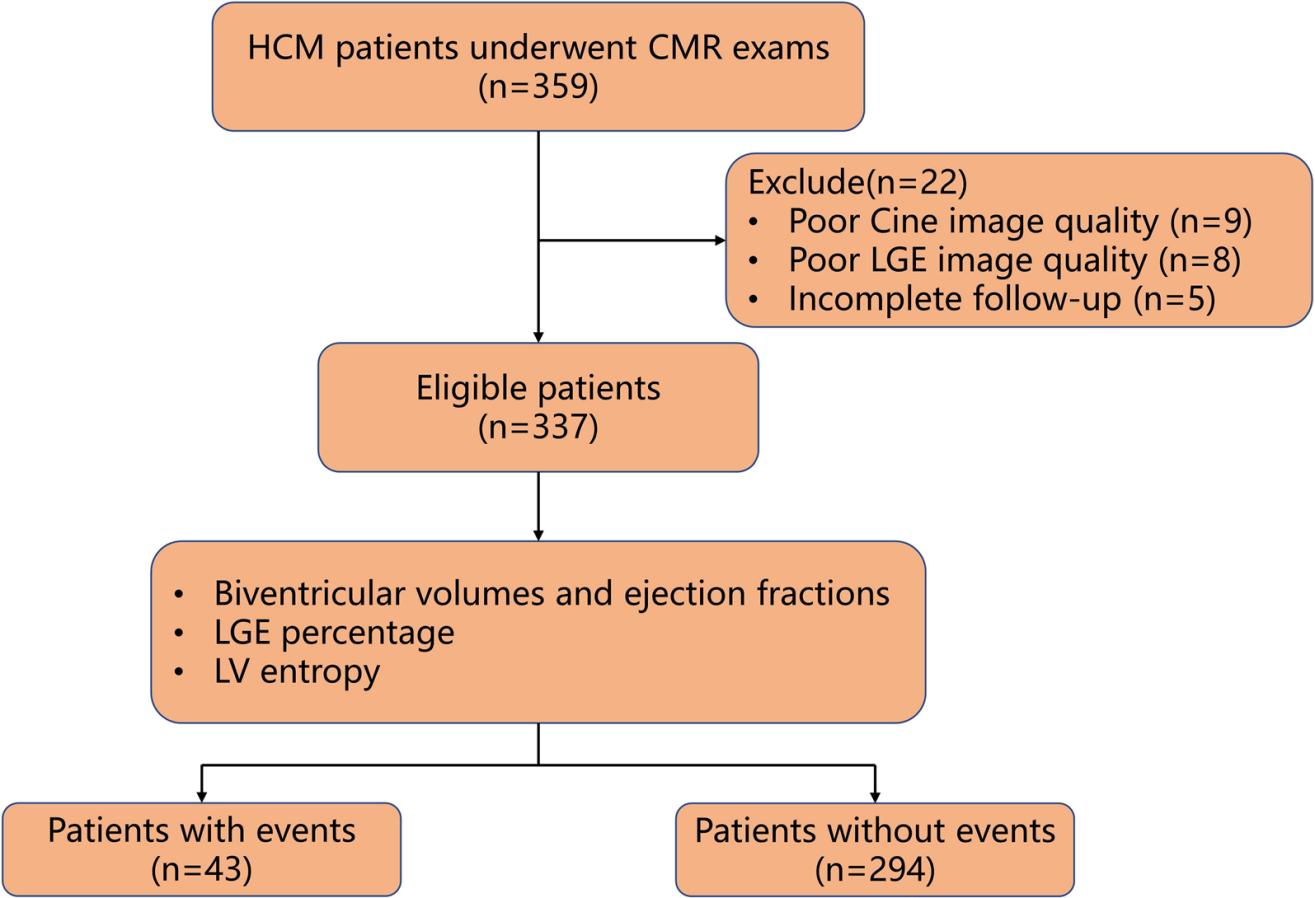
Flowchart of numbers of patients recruited in the study

This retrospective study was approved by the local hospital ethics committees, and written informed consent was obtained from all the patients. HCM is diagnosed by CMR confirming the presence of non-dilated LV hypertrophy (maximal wall thickness ≥ 15 mm in adult patients or ≥ 13 mm in relatives of adult patients) with the absence of another disease that could explain LV hypertrophy [23]. A total of 359 patients with HCM were recruited to undergo CMR between May 2017 and December 2020, of which 337 patients with adequate CMR sequences and acceptable image quality were included in the later follow-up. The exclusion criteria were as follows: (a) failure to complete cardiac magnetic resonance; (b) poor image quality; (c) incomplete follow-up. Other exclusion criteria included congenital heart disease, myocardial amyloidosis, advanced renal failure, or contraindication to gadolinium-based contrast agents (Fig. 1).

### CMR protocol

CMR studies were conducted on 3.0 T scanners (Magnetom Verio; Siemens AG Healthcare, Germany and Ingenia; Philips Healthcare, the Netherlands). The sequences included steady-state free precession breath-hold cine images and LGE images. The detailed CMR protocols are presented in Additional file 1.

### CMR image analysis

The CMR image analysis was performed using CVI42 (Circle Cardiovascular Imaging Inc.) by two radiologists with more than 5 years of experience who were blinded to the clinical information. The LV endocardial and epicardial borders were automatically delineated and manually adjusted at end-systole and end-diastole. Then, the LV end systolic volume (LVESV), LV end-diastolic volume (LVEDV), LV mass (LVM) and LV ejection fraction (LVEF) were calculated by the software. All volumes and masses were normalized to the body surface area (BSA). The percentage of LGE was identified and calculated by CVI software with a full width at half-maximum (FWHM) method.

### LV entropy measurement

**Fig. 2:**
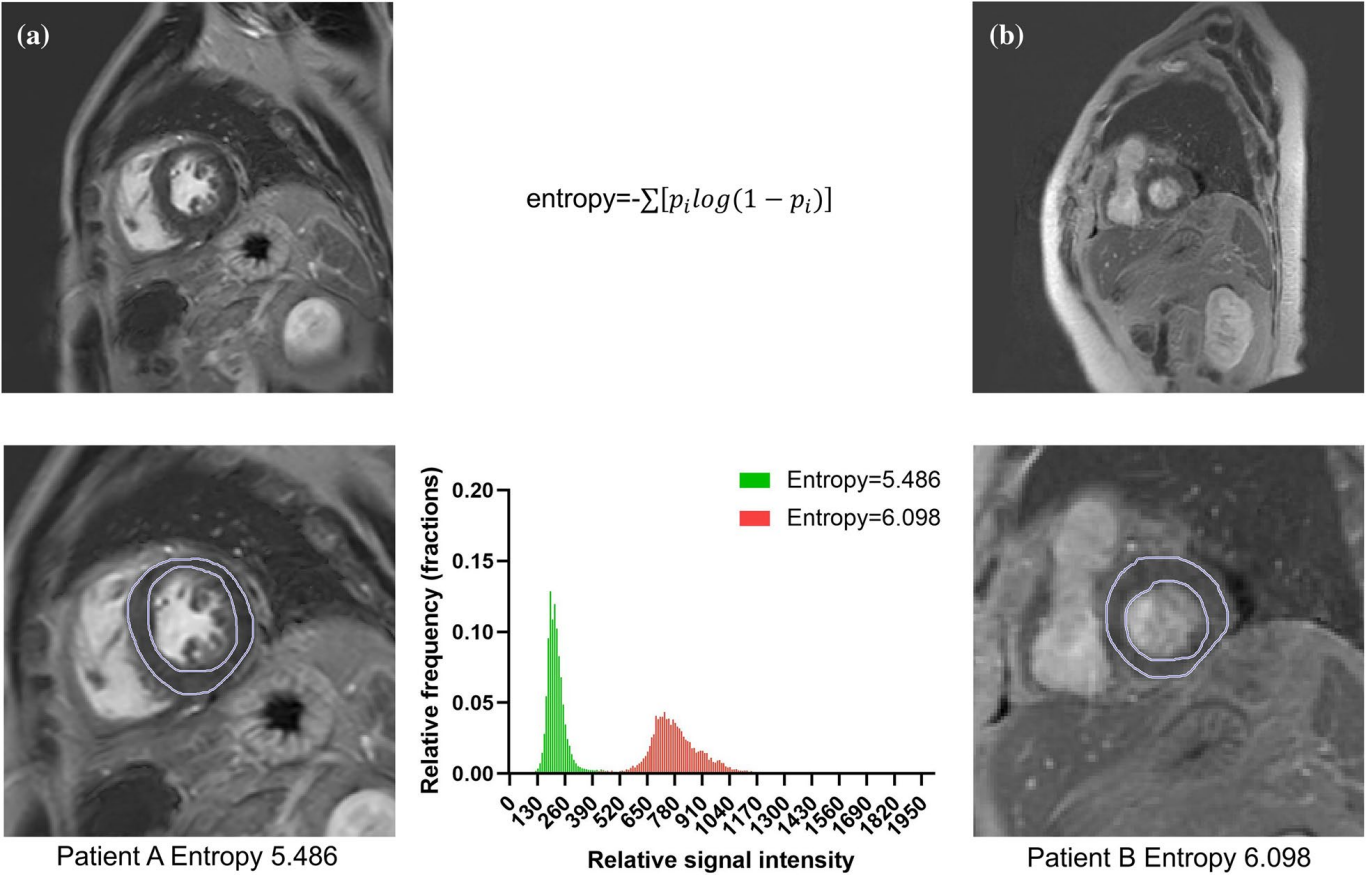
Schematic diagram of the left ventricular (LV) entropy measurement. The LV entropy was obtained by calculating the distribution of the pixel signal intensities of the myocardial on LGE images. The epicardial and endocardial borders were manually traced, and excluded the blood pool signal. The software automatically calculated the signal intensity of each pixel of the myocardium. The histogram shows the probability distribution of the pixel signal intensities for patient A and patient B

The LV entropy was obtained by calculating the distribution of the pixel signal intensities of the myocardial on LGE image (Fig. 2). The epicardial and endocardial borders were manually traced on the LGE images and excluded the blood pool signal. A program written in MatLab (MathWorks, Natick, MA) automatically performed the entropy calculation according to the following equation:

$$\mathrm{entropy}=-\sum \left[{p}_{i}log(1-{p}_{i})\right]$$

where ${p}_{i}$ is the probability distribution of signal intensity.

### Follow-up information

The follow-up data were collected by two cardiologists with more than ten years of experience via medical records and telephone interviews who were unknown to the CMR data. The primary endpoint was defined as readmission to the hospital owing to HF. The secondary endpoints comprised the primary endpoint, sudden cardiac death and non-cardiovascular death.

### Statistical analyses

Data were analyzed by IBM SPSS statistics software (v. 24.0, IBM SPSS Inc.) and R software (version 4.1.2; The R Project for Statistical Computing). T test and Chi-square test were used to compare the continuous variables (presented as mean ± SD) and categorical variables (presented as frequencies with percentages). Correlations between continuous variables were assessed using Pearson's correlation coefficient. The survival curves were established according to the Kaplan–Meier method and high and low entropy patients were classified by median. Using Cox regression analyses to determine whether entropy was a predictor of events. Significant variables (*p* < 0.1) from univariate regression analysis were included in the multivariate analysis. To assess the incremental prognostic value of LGE entropy, we calculated the Harrel C-index, the net reclassification improvement (NRI), and the integrative discrimination index (IDI). The intraclass correlation efficient was computed to evaluate the intra- and inter-observer agreement.

## Results

### Study population characteristics

A total of 337 patients formed the cohort for this analysis. Table 1 shows the clinical and CMR characteristics of HCM patients. Meanwhile, 43 patients (mean age 54 ± 15 years, 84% male) with endpoint events had a higher BMI, HR, and left atrium (LA) diameter (all *p* < 0.05). Regarding CMR parameters, patients with HCM who reached the endpoint events had significantly higher LVEDVi (89.3 ± 40.5 ml/m^2^), LVESVi (47.6 ± 21.2 ml/m^2^), LGE (13.9 ± 10.0%) and entropy (6.20 ± 0.45), and lower LVEF (43.0 ± 11.8%) and SV (56.5 ± 29.8 ml) than those patients who did not have endpoint events (all *p* < 0.05). The intra-observer and inter-observer agreements showed good reproducibility of entropy measurements (0.965 and 0.943, *p* < 0.05 for both). There was no statistical difference in the acquisition of entropy by both scanners between normal patients and between patients who reached the endpoint events (Additional file 1: Table S3).

**Table 1:** Basic information and CMR characteristics between patients with and without events

Characteristics	No events (n = 294)	Events (n = 43)	*p* value
*Demographics*			
Age (y)	51 ± 14	54 ± 15	.196
Male	217 (74)	36 (84)	.161
BMI (kg/m^2^)	24.9 ± 1.6	25.5 ± 1.1	.001
BSA (m^2^)	1.8 ± 0.2	1.9 ± 0.1	.107
SBP (mm Hg)	120 ± 9	117 ± 19	.374
DBP (mm Hg)	78 ± 6	77 ± 11	.688
HR (beats/min)	73 ± 9	79 ± 14	.006
Hypertension	57 (19)	12 (28)	.196
Diabetes	27 (9)	4 (9)	.980
CAD	22 (8)	3 (7)	.906
Unexplained syncope	24 (7)	6 (11)	.638
SCD family history	28 (10)	7 (12)	.607
NSVT	19 (7)	5 (9)	.595
NYHA functional class			
I	210 (71)	0 (0)	
II	31 (11)	8 (19)	
III	30 (14)	4 (9)	
IV	13 (4)	31 (72)	
LA diameter (mm)	41.2 ± 7.8	44.8 ± 9.6	.006
Maximal LVWT (mm)	19.5 ± 12.7	18.3 ± 4.4	.522
LVOT gradient pressure (mm Hg)	17.5 ± 12.2	17.1 ± 12.6	.816
CMR parameters			
LVEF (%)	66.3 ± 12.1	43.0 ± 11.8	< .001
LVEDVi (ml/m^2^)	63.1 ± 22.6	89.3 ± 40.5	< .001
LVESVi (ml/m^2^)	34.6 ± 12.6	47.6 ± 21.2	< .001
SV (ml)	77.7 ± 49.4	56.5 ± 29.8	.007
LV mass index (g/ m^2^)	90.7 ± 41.1	102.8 ± 38.4	.071
LGE (%)	5.0 ± 5.3	13.9 ± 10.0	< .001
LV wall entropy	5.59 ± 0.35	6.20 ± 0.45	< .001

Data are mean ± SD or n (%)

Events = composite of primary and secondary endpoints

The primary endpoint defined as readmission to the hospital owing to heart failure

The secondary endpoint was the composite of the primary endpoint, sudden cardiac death and non-cardiovascular death

In addition, the correlation of entropy with characteristic LV parameters in patients with HCM is shown in Additional file 1: Table S1. In all HCM patients, entropy showed a significant positive correlation with LA diameter, LVEDVi, LVESVi, and LGE (all *p* < 0.05). Significant negative correlations were observed with LVEF and SV (all *p* < 0.05).

### Outcome

**Fig. 3:**
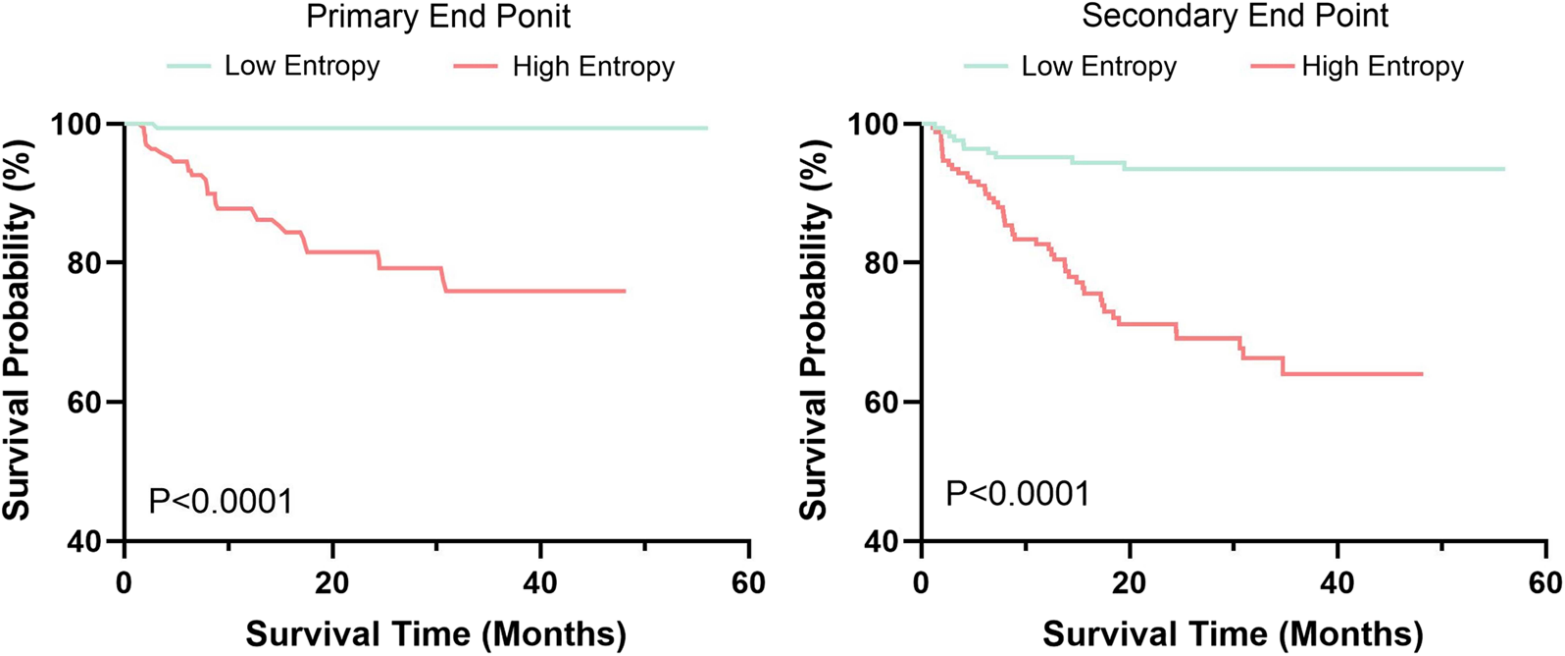
Kaplan–Meier curves for primary and secondary endpoints in patients with hypertrophic cardiomyopathy according to the median of LGE entropy (high entropy group ≥ 5.587 and low entropy < 5.587)

During a median follow-up of 24 ± 13 months, 33 (10%) patients had reached the primary endpoint event. Moreover, 43 (13%) patients reached the secondary endpoint event, including the primary endpoint (10%), eight (2%) with cardiovascular death and two (1%) with non-cardiovascular death. Kaplan–Meier analysis (Fig. 3) demonstrated that patients with high entropy greater than or equal to the median (≥ 5.587) had a significantly higher risk of outcome events than patients with low entropy (< 5.587) (*p* < 0.001 for both).

### Survival analysis

Classical risk factors from the 2020 AHA/ACC guidelines including age, unexplained syncope, non-sustained ventricular tachycardia (NSVT), SCD family history, LA diameter, maximal LV wall thickness, left ventricular outflow tract (LVOT) gradient pressure, and LGE were included in the Cox regression analysis. Univariate Cox analysis showed age (HR 1.026, CI 0.999–1.054), LA diameter (HR 1.004, CI 0.999–1.008), LGE (HR 1.107, CI 1.074–1.142) and entropy (HR 1.356, CI 1.258–1.463) were significant predictors of the primary endpoint events (all *p* < 0.1, Table 2). LA diameter (HR 1.005, CI 1.002–1.009), LGE (HR 1.116, CI 1.089–1.144) and entropy (HR 1.356, CI 1.276–1.441) were significant predictors of the secondary endpoints (all *p* < 0.1). In addition, analysis of entropy with each adverse event showed that entropy was a significant univariate predictor of each event (all *p* < 0.001, Additional file 1: Table S2).

**Table 2:** Univariable cox regression analysis for outcomes in the HCM cohort

Characteristics	Primary end point (n = 33)		Secondary end point (n = 43)	
	Unadjusted hazard ratio	*p* value	Unadjusted hazard ratio	*p* value
Age	1.026 (0.999, 1.054)	.055	1.016 (0.994, 1.038)	.156
Unexplained syncope	0.044 (0.001, 15.026)	.294	1.918 (0.809, 4.548)	.139
NSVT	0.518 (0.071, 3.805)	.518	1.551 (0.553, 4.347)	.404
SCD family history	0.636 (0.152, 2.664)	.535	0.937 (0.335, 2.622)	.901
LA diameter	1.004 (0.999, 1.008)	.088	1.005 (1.002, 1.009)	.002
Maximal LVWT	0.936 (0.861, 1.016)	.114	0.968 (0.906, 1.035)	.344
LVOT gradient pressure	0.980 (0.950, 1.011)	.200	0.994 (0.970, 1.019)	.653
Maximal LVWT ≥ 30 mm	0.048 (0.001, 652.152)	.531	0.833 (0.115, 6.053)	.857
LGE	1.107 (1.074, 1.142)	< .001	1.116 (1.089, 1.144)	< .001
LV wall entropy	1.356 (1.258, 1.463)	< .001	1.356 (1.276, 1.441)	< .001

Significant variables were included in multivariate Cox regression analysis. The collinearity analysis of LGE and entropy excluded the collinearity [Tol = 1 (> 0.1) and VIF = 1 (< 10)]. In multivariate Cox analysis, LGE (HR 1.048, CI 1.008–1.090) and entropy (HR 1.291, CI 1.182–1.411) were significant predictors of the primary endpoint (all *p* < 0.05, Table 3). For secondary endpoint events, LGE (HR 1.055, CI 1.021–1.091) and entropy (HR 1.273, CI 1.183–1.370) were significant predictors (all *p* < 0.05).

**Table 3:** Multivariable cox regression analysis for outcomes in the HCM cohort

Characteristics	Primary end point (n = 33)		Secondary end point (n = 43)	
	Unadjusted hazard ratio	*p* value	Unadjusted hazard ratio	*p* value
Age	1.015 (0.989, 1.042)	.262	1.004 (0.982, 1.027)	.703
Unexplained syncope				
NSVT				
SCD family history				
LA diameter	0.999 (0.994, 1.004)	.795	1.002 (0.998, 1.006)	.425
Maximal LVWT				
LVOT gradient pressure				
Maximal LVWT ≥ 30 mm				
LGE	1.048 (1.008, 1.090)	.018	1.055 (1.021, 1.091)	.001
LV wall entropy	1.291 (1.182, 1.411)	< .001	1.273 (1.183, 1.370)	< .001

### Prediction models

**Fig. 4:**
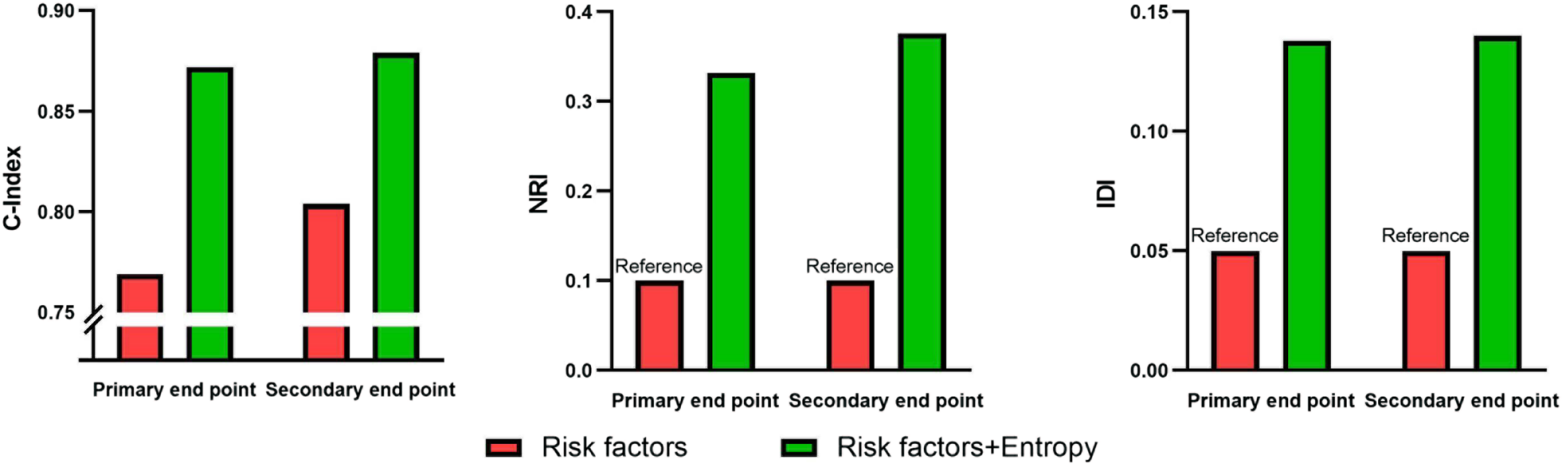
Evaluation of the accuracy and improvement of LGE entropy

We evaluated the level of improvement in the prediction model after adding entropy to the existing risk factors including age, LA diameter and LGE (Table 4 and Fig. 4). For both primary and secondary endpoints, the C-index (0.872 and 0.838) were improved with the addition of entropy. Discrimination and reclassification to predict the occurrence of primary and secondary endpoint events (NRI 0.332 and 0.376, IDI 0.138 and 0.140) were also significantly improved with the addition of entropy (*p* < 0.05).

**Table 4:** Evaluation of the accuracy and reclassification of LV entropy

	Primary end point		
	C-index (95% CI)	NRI (95% CI)	IDI (95% CI)
Model 1 (risk model + LGE)	0.769 (0.693, 0.844)	Baseline	Baseline
Model 2 (risk model + LGE + entropy)	0.872 (0.821, 0.924)	0.332 (0.190, 0.642)	0.138 (0.020, 0.288)

	Secondary end point		
	C-index (95% CI)	NRI (95% CI)	IDI (95% CI)
Model 1 (risk model + LGE)	0.804 (0.739, 0.870)	Baseline	Baseline
Model 2 (risk model + LGE + entropy)	0.879 (0.838, 0.919)	0.376 (0.007, 0.662)	0.140 (0.012, 0.269)

## Discussion

This study showed that LGE entropy was significantly higher in HCM patients with adverse events. Moreover, LGE entropy was significantly correlated with LA diameter, LVEF and LGE. In the survival analysis, entropy > 5.587 was found to be associated with primary and secondary endpoints. In multivariate Cox regression analysis, LGE entropy was also an independent predictor of primary and secondary endpoints. These findings suggested that LGE entropy provides incremental prognostic value for the prediction of adverse events in patients with HCM.

Despite effective advances in the prevention of adverse events such as SCD, the effective identification of adverse prognosis in patients with HCM remains unsatisfactory, mainly because of the significant heterogeneity in clinical phenotypes, with heart failure being the main complication determining the long-term prognosis of HCM [24–26]. Treatment of HCM has improved significantly in the last few decades with medications, myectomy, alcohol septal ablation, and heart transplantation, but inadequate identification of high-risk patients affects the long-term prognosis of patients [28].

CMR has played an increasingly important role in the evaluation of HCM patients. CMR using LGE to detect myocardial fibrosis is the most widely studied technique to non-invasively characterize underlying scar structures [29]. It has been found that half to two-thirds of patients with HCM may have LGE, and fibrosis is the leading cause of cardiac dysfunction in patients with cardiomyopathy [30–32]. A recent meta-analysis of five studies demonstrated that the presence of LGE was associated with a 3.4 fold increase in risk for sudden cardiac death (SCD), 1.8 fold increase in all-cause mortality, 2.9 fold increase in cardiovascular mortality and an increasing trend in HF death [16]. Similar to previous studies, LGE was more significant in patients with adverse outcomes in this study, and LGE was also a significant predictor of endpoint events.

However, LGE quantitation in clinical examination focuses on detecting the presence and extent of scars and has limitations in the assessment of the overall myocardium, especially the non-enhanced areas [17]. HCM is characterized by diffuse histopathological abnormalities, with lesions often involving the entire LV myocardium [22]. Patients with HCM may develop more than three types of myocardial fibrosis during the disease: diffuse micro-scars, perivascular fibrosis, perimysial, and endomysial fibrosis [33, 34]. The heterogeneity between the different fibrosis may also lead to different degrees of myocardial structural abnormalities and lead to adverse events finally [35]. Therefore, patients with HCM need to be differentiated more carefully in terms of myocardial tissue heterogeneity including normal myocardium and fibrotic scars. Entropy, a measure of image complexity, has been widely used in recent years in CMR to assess myocardial heterogeneity [18, 20, 21, 36]. Low entropy indicates a uniformity of pixel intensities, reflecting the same type of tissue in the myocardium. While high entropy indicates that regional signal intensity values have an irregularly wide range, which reflects the presence of a mixture of different tissue types [37]. In contrast to LGE based on visual and signal intensity thresholds for myocardial scar assessment, entropy measurements are designed to capture tissue heterogeneity throughout the left ventricle. Muthalaly et al. [21] first applied the concept of entropy to the field of CMR and proved to be a fast and reproducible measure, and entropy contributed to risk stratification in patients with dilated heart disease. Androulakis et al. [20] found that in post-MI patients, whole LV entropy was independently associated with mortality by reflecting poor and irreversible inhomogeneous remodeling of the LV after infarction. Antiochos et al. [18] found that LV entropy became the strongest multivariate predictor of MACE in patients with arrhythmias without myocardial scarring.

In this study, patients with elevated LGE entropy had a higher incidence of HF, which is consistent with the results of Antiochos et al. [18], where entropy detection could improve the prediction of HF risk by effectively identifying plaque-like fibrosis and coarser fibrosis strands that are not easily detected by LGE. The association between LGE entropy and HF may reflect the level of inhomogeneous fibrosis in HCM patients. In addition, LGE entropy also improved the predictive accuracy of predictive models for outcome events in terms of comparison of predictive models. In addition to the C-index, the results of NRI and IDI calculations also showed that LGE entropy has higher predictive power for endpoint events including HF.

This study had several limitations. First, due to the limitation of patient numbers, the incidence of endpoints events is higher compared to previous large sample researches. We will continue to recruit more cases to refine the analysis in the future. The measurement of LGE entropy also needs further generalization studies on more devices. Second, T1 mapping was not performed in most patients in this study, and future comparison of entropy with extracellular volume fraction is needed for more reliable validation. Given the changes in HCM patients with clinical treatment, multiple measurements of entropy during treatment would help to make more accurate analytical judgments.

In conclusion, the use of LGE entropy measurements facilitates analysis of myocardial heterogeneity in HCM patients. LGE entropy has independent prognostic value for poor prognosis in patients with HCM. The inclusion of LGE entropy as a risk factor provides incremental prognostic value.

### Supplementary Information

**Additional file 1. **Late gadolinium enhancement entropy as a new measure of myocardial tissue heterogeneity for prediction of adverse cardiac events in patients with hypertrophic cardiomyopathy.

## Data Availability

The data used in the study are available from the corresponding author on reasonable request.

## Abbreviations

BMI: Body mass index
BSA: Body surface area
CMR: Cardiac magnetic resonance
HCM: Hypertrophic cardiomyopathy
HF: Heart failure
HR: Heart rate
IDI: Integrative discrimination index
LGE: Late gadolinium enhancement
LV: Left ventricular
LVEDV: LV end-diastolic volume
LVEF: LV ejection fraction
LVESV: LV end systolic volume
LVM: LV mass
NRI: Net reclassification improvement
SCD: Sudden cardiac death
