# Supplementary material for: Late gadolinium enhancement entropy as a new measure of myocardial tissue heterogeneity for prediction of adverse cardiac events in patients with hypertrophic cardiomyopathy
Source: Insights Imaging. 2023 Aug 21;14:138. doi: 10.1186/s13244-023-01479-6 (PMC10441833; doi:10.1186/s13244-023-01479-6)
Supplement: Supplementary file 1 — Additional file 1. Late gadolinium enhancement entropy as a new measure of myocardial tissue heterogeneity for prediction of adverse cardiac events in patients with hypertrophic cardiomyopathy. [file 13244_2023_1479_MOESM1_ESM.pdf]

Late Gadolinium Enhancement Entropy as a New Measure of Myocardial Tissue Heterogeneity for Prediction of Adverse Cardiac Events in Patients with Hypertrophic Cardiomyopathy

ELECTRONIC SUPPLEMENTARY MATERIAL

**Supplementary Table 1: Association of LV Entropy with LV Characteristics in Participants with HCM**		
**Variable**	**r**	**P**
LA diameter (mm)	.189	<.001
Maximal LVWT (mm)	.044	.420
LVOT gradient pressure (mm Hg)	.042	.439
LVEF %	-.468	<.001
LVEDVi (ml/m2)	.240	<.001
LVESVi (ml/m2)	.260	<.001
SV (ml)	-.116	.033
LV mass index (g/ m2)	.099	.068
LGE (%)	.487	<.001

**Supplementary Table 2: Univariate association of LV wall entropy with each end point event.**		
	**HR (95% CI)**	**P value**
**Heart Failure Hospitalization**		
LV wall entropy	1.356 (1.258, 1.463)	<.001
**Sudden Cardiac Death (SCD)**		
LV wall entropy	1.332 (1.183, 1.499)	<.001
**Non-cardiovascular Death**		
LV wall entropy	1.354 (1.222, 1.501)	<.001

**Supplementary Table 3: Comparison of the entropy obtained by the two scanners.**			
	scanner1 (n=106)	scanner2 (n=188)	P
normal patients	5.61±0.31	5.57±0.37	0.315
	scanner1 (n=14)	scanner2 (n=22)	P
endpoints-patients	6.19±0.56	6.22±0.44	0.865

Supplementary Figure 1: Box plots of LVEF, LGE and Entropy.

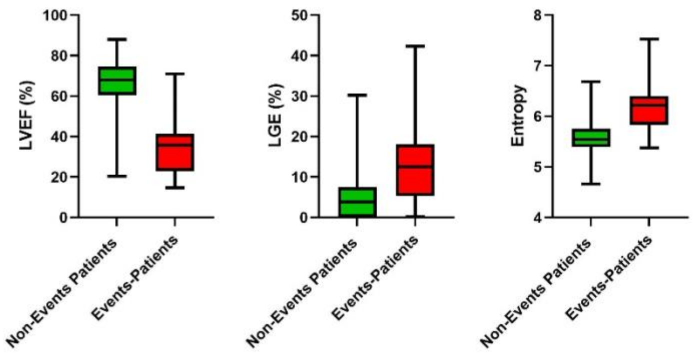

Supplemental material section of CMR scan protocols

CMR studies were conducted on 3.0 T scanners (Magnetom Verio; Siemens AG Healthcare, Germany and Ingenia; Philips Healthcare, the Netherlands). The standardized imaging protocol consisted of steady-state free precession breath-hold cine images and late-enhanced images (LGE) using gadolinium. Steady-state free-precession cine images were obtained during repeated breath-holds in three long axes (2-chamber, 3-chamber, and 4-chamber view) and in a stack of short axes covering the LV. A commercially available gadolinium-based contrast agent (gadopentetate dimeglumine, Magnevist, Bayer Healthcare) was administered intravenously at a dose of 0.1 mmol/kg body weight, and contrast enhanced images were acquired after a 10-minute delay with the use of an inversion recovery segmented gradient echo sequence The Magnetom Verio was equipped with a 32-channel cardiac coil. The parameters included: (1) b-SSFP cine sequences: TR: 43.5 ms, TE: 1.45 ms, slice thickness = 8 mm. FOV: 300×300 mm. acquired matrix = 1.25 mm x 1.25 mm. （2）LGE images TR: 900 ms; TE: 3.5 ms. slice thickness = 8 mm. FOV: 300×300 mm. acquired matrix = 0.98 mm x 0.98 mm.

The Ingenia was equipped with a 12-element phased-array coil. The parameters included: (1) b-SSFP cine sequences: repetition time (TR) = 2.8 ms, echo time (TE) = 1.4 ms, slice thickness = 7 mm, acquired matrix = 1.2 mm x 1.2 mm, phases per cardiac cycle = 30, field of view (FOV) = 300 mm x 300 mm. （2）PSIR sequence: TR = 6.1 ms, TE = 3 ms, slice thickness = 10 mm, FOV = 300 mm x 300 mm, acquired matrix = 1.6 mm x 1.9 mm.
